# Reconciling Counterintuitive Findings and Quantifying Potential Sources of Bias in Cognitive Aging Research

**DOI:** 10.1093/geroni/igaf122.536

**Published:** 2025-12-31

**Authors:** Emma Nichols, Aleda Leis, Lindsay Kobayashi

## Abstract

Epidemiologic research on cognitive aging poses methodological challenges including survival bias, measurement error, and unmeasured confounding. The application of novel methodological approaches can help quantify sources of bias and reconcile counterintuitive findings to advance our understanding of the causes and consequences of cognitive decline and dementia. This session features innovative research on cognitive aging using a wide range of methodologies, including latent growth curve modeling, inverse probability weighting, assessments of potential sources of measurement error, and simulation analysis to better understand the magnitude and role of biasing factors in cognitive aging research. First, Dr. Zachary Kunicki will estimate the rate of normative cognitive changing, addressing potential biases in prior research by using the long follow-up and narrow age range available in the Children of the Depression Age (CODA) cohort, and accounting for mode and practice effects. Second, Dr. Michelle Shardell will evaluate potential reasons for the observed inverse association between cancer and dementia by using inverse probability weights to address selective attrition and data on plasma metabolomics to assess unmeasured confounding through shared biological mechanisms. Third, Dr. Yezhen Li will discuss the potential for measurement error due to noise during cognitive testing and how this may vary by hearing impairment. Finally, Dr. Ryan Andrews will estimate the impact of pre-enrollment survival bias across different country contexts in cross-national research using simulation analysis. Dr. Lindsay Kobayashi will serve as the discussant, integrating findings to discuss the benefits of quantifying sources of bias and implications for future research in cognitive aging.

This is a collaborative symposium between the Epidemiology of Aging and Measurement, Statistics, and Research Design Interest Groups.

Epidemiology of Aging Interest Group Sponsored Symposium

